# FRACTURE MRI: evaluation of imaging capability in hand tendon visualization using healthy volunteer MRI

**DOI:** 10.1186/s13244-025-02182-4

**Published:** 2026-01-12

**Authors:** Yukari Matsuzawa, Yusuke Matsuura, Kaoru Kitsukawa, Hajime Fujimoto, Hiroki Mukai, Jun Hashiba, Takafumi Yoda, Ryuna Kurosawa, Takayuki Sada, Yoshihito Ozawa, Yuki Shiko, Kohei Takahashi, Takahiro Yamazaki, Kayo Inaguma, Takane Suzuki, Seiji Ohtori

**Affiliations:** 1 Department of Orthopaedic Surgery, Graduate School of Medicine, Chiba University, Chiba, Japan; 2 Comprehensive Radiology Center, Chiba University Hospital, Chiba, Japan; 3 Department of Radiology, Chiba University Hospital, Chiba, Japan; 4 Biostatistics Section, Clinical Research Center, Chiba University Hospital, Chiba, Japan; 5 Department of Biostatistics, Graduate School of Medicine, Saitama Medical University, Saitama, Japan

**Keywords:** FRACTURE, VR image, Hand and finger tendons, Visualization

## Abstract

**Objectives:**

To evaluate the conspicuity of fast field echo resembling a CT using restricted echo-spacing (FRACTURE) in visualizing hand tendons and assess the utility of FRACTURE-derived volume rendering (VR) images using MRI in healthy individuals.

**Materials and methods:**

This prospective observational study enrolled ten healthy volunteers who underwent MRI, including FRACTURE, three-dimensional proton density-weighted volume isotropic turbo spin-echo acquisition (PD-VISTA), and two-dimensional T2-weighted image (T2WI) in neutral and ulnar deviation positions. VR images depicting bones and tendons were created from FRACTURE data. Twenty-four flexor and extensor tendons were qualitatively evaluated by four experienced readers using a 5-point scale for cross-sectional images (including FRACTURE inversion) and a 3-point scale for VR images. Quantitative analysis included tendon cross-sectional area measurements and contrast-to-noise ratio (CNR) calculations. Inter- and intra-reader reliability and FRACTURE-inversion agreement were assessed using weighted kappa coefficients. Statistical analysis included an ordinal mixed-effects model, Bland–Altman analysis, correlation coefficients, and paired *t*-tests.

**Results:**

Ten healthy volunteers (5 men, 5 women, mean age 37.4 ± 9.1 years) were evaluated. FRACTURE achieved the highest qualitative scores (3.30 ± 0.364) compared to PD-VISTA (3.09 ± 0.265) and T2WI (2.60 ± 0.509), showing statistically significant superiority by ordinal mixed-effects modeling (*p* < 0.001). FRACTURE inversion showed high agreement with FRACTURE (weighted kappa = 0.975). Tendon cross-sectional area measurements showed strong correlations between sequences (*r* = 0.680–0.740) but significant systematic bias (*p* < 0.017), with FRACTURE measuring consistently smaller areas. FRACTURE demonstrated significantly higher CNR for muscle-tendon comparisons (12.63 ± 1.088 vs 7.911 ± 1.746, *p* < 0.017).

**Conclusion:**

FRACTURE provides superior hand tendon visualization compared to conventional MRI sequences, with potential value for clinical practice.

**Critical relevance statement:**

FRACTURE showed superior hand tendon visualization compared to T2WI and PD-VISTA, potentially helping assess anatomical variations. VR images provide a three-dimensional understanding of the hand tendon structure. These capabilities could enhance surgical planning and procedure selection in hand surgery.

**Key Points:**

- FRACTURE performs better than T2WI and PD-VISTA for evaluating hand tendons.
- FRACTURE provides excellent contrast, enabling the creation of VR images.
- FRACTURE could serve as an aid in surgical planning and procedure selection, with the potential to improve hand surgery practice.

**Graphical Abstract:**

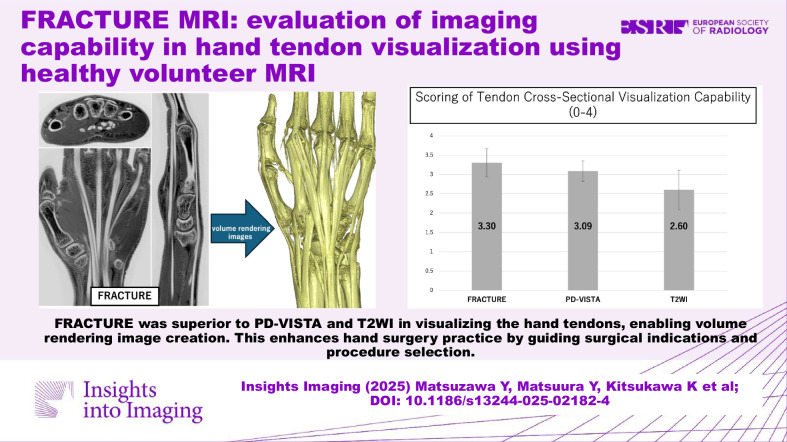

## Introduction

Tendon injuries of the hand, including traumatic injuries and subcutaneous ruptures associated with degenerative joint diseases, are commonly encountered in clinical practice. The standard treatment involves surgical intervention, such as tendon suturing, grafting, and transfer. An accurate preoperative assessment of the tendon injury site and the condition of the donor tendon is important. However, hand tendons, particularly extensor tendons, often exhibit anatomical variations [1–6]. The extensor indicis proprius (EIP) tendon, frequently utilized in tendon transfer procedures, is typically inserted on the ulnar side of the metacarpophalangeal (MP) joint of the index finger. Nevertheless, variations in its insertion, course, and number of slips are common, with cases of hypoplasia or absence reported [1, 2]. Additionally, variations in the course of the extensor digitorum communis (EDC) tendon [3] and anatomical diversity of the abductor pollicis longus tendon and extensor pollicis brevis (EPB) tendon have been reported [4]. Moreover, the presence of anomalous muscles [5, 6] further emphasizes the anatomical variability of hand tendons. The precise preoperative identification of these anatomical variations facilitates smoother surgical procedures.

Conventional diagnostic methods for tendon injuries include ultrasonography, and magnetic resonance imaging (MRI). Ultrasonography has been reported to excel in evaluating injured tendon sites [7] but is operator-dependent and lacks comprehensive 3D spatial resolution. MRI is one of the most effective modalities for assessing both the anatomical configuration and structural integrity of wrist tendons. Injured tendons show increased water content and disrupted collagen fiber arrangement, causing prolonged T2 values that are easily visualized with conventional MRI [8]. However, normal tendons exhibit extremely short T2 values [9, 10], causing rapid proton signal decay in conventional MRI sequences. This results in decreased signal intensity, making the detection of subtle anatomical variations in normal tendons difficult. Furthermore, comprehensively understanding the three-dimensional configuration of finger tendons remains challenging, necessitating more convenient imaging methods.

Fast-field echo resembling a CT using restricted echo-spacing (FRACTURE), a CT-like bone contrast sequence from Philips Healthcare, is based on a three-dimensional gradient recalled echo technique and was developed to provide high sensitivity for depicting tissues with extremely short T2 relaxation times, such as bone [11]. FRACTURE applies grayscale inversion processing to the acquired images, rendering low-signal structures such as cortical bone and tendons as bright (white), while displaying surrounding fat (high signal) and muscle (intermediate signal) as relatively dark. This creates high contrast like CT images, enabling clear delineation of tissue contours [12]. Tendons, characterized by densely oriented collagen fibers, represent a typical example of short T2 tissues. Therefore, we considered that FRACTURE has the potential to visualize tendon structures with clarity comparable to bone imaging. We also found that FRACTURE sequences have the technical capability to create volume rendering (VR) images that can produce atlas-like anatomical images clearly depicting tendon morphology, course, and insertion sites.

There are no previous reports on tendon visualization using FRACTURE, and its capability remains unclear. Additionally, the subjective clarity of tendon visualization in FRACTURE-derived VR images has not been elucidated. We hypothesized that FRACTURE provides superior tendon visualization compared to conventional sequences. This study aimed to evaluate FRACTURE’s conspicuity in visualizing hand tendons and assess subjective clarity of tendon depiction in FRACTURE-derived VR images using healthy volunteer MRI.

## Materials and methods

### Study design

This prospective observational study enrolled ten healthy volunteers who underwent imaging from October 2022 to March 2023. Participants were selected with consideration to avoid bias in gender and age distribution. Those with a history of hand injury or medical conditions that could affect hand and tendon function, including rheumatoid arthritis, collagen diseases, autoimmune disorders, and other inflammatory conditions, were excluded. The institutional ethics committee approved the study (approval number: M10453), and all participants provided informed consent.

### MRI acquisition protocol

**Fig. 1:**
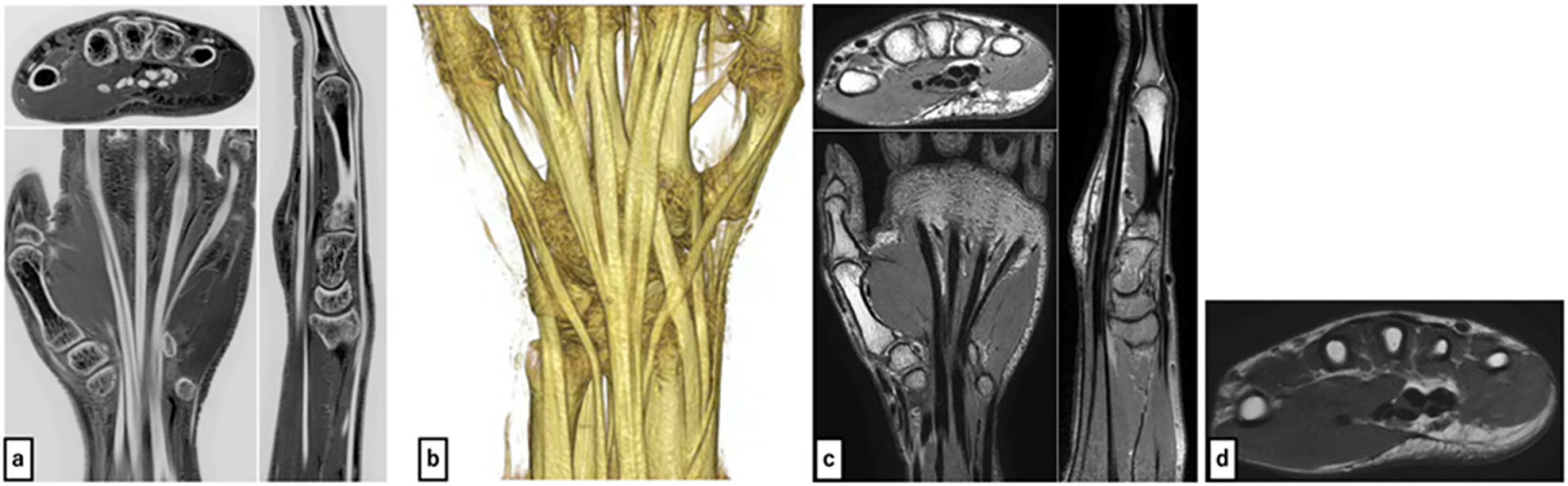
**a** FRACTURE sequence (axial, coronal, and sagittal reformation) shows tendons as distinct high signal intensity similar to cortical bone, with clear contrast against surrounding musculature. **b** Volume-rendered image from the FRACTURE sequence depicts the tendon course alongside skeletal structures, facilitating easy assessment of an individual patient’s tendon conditions. **c** PD-VISTA sequence (axial, coronal, and sagittal reformation) shows tendons as low signal intensity structures and are relatively clearly visualized. **d** Axial T2-weighted imaging sequence depicts tendons as low signal intensity structures with relatively clear visualization

Imaging was performed using a 3.0-Tesla MRI unit (Intera Achieva Nova Dual Release 5, Philips Medical Systems) with a dStream Small Extremity 8ch coil. FRACTURE, an isotropic three-dimensional imaging method, was conducted. For comparison, three-dimensional proton density-weighted volume isotropic turbo spin-echo acquisition (PD-VISTA) and two-dimensional T2-weighted imaging (T2WI) were also obtained. Imaging parameters are shown in Table 1, with representative images in Fig. 1.

**Table 1:** Sequence protocol

	FRACTURE	PD-VISTA	T2WI
Image orientation	Coronal	Coronal	Axial
Repetition time [ms]	18	1300	5000
Echo time[ms]	2.3/4.6/6.9/9.2	34	80
Flip angle [degrees]	15	90	90
Echo train length	n/a	45	15
Number of signals acquired	1	1	2
Section thickness [mm]	0.6	0.6	3.0
Resolution (reconstruction) [mm^3^]	0.6/0.6/0.6 (0.3/0.3/0.3)	0.6/0.6/0.6 (0.3/0.3/0.3)	0.3/0.3/3.0 (0.2/0.2/3.0)
Field of view [mm^2^]	200	200	160
Acquisition matrix	332 × 264	332 × 250	532 × 415
Bandwidth [Hz/pixel]	558	339	287
Acquisition time (min:s)	3 min 32 s	3 min 46 s	3 min 40 s

Each sequence was performed in neutral position (0° wrist extension, 0° ulnar deviation, and finger extension) and ulnar deviation position (0° wrist extension, maximum ulnar deviation, and finger extension). Considering the influence of the magic angle effect described below, imaging was also performed in the ulnar deviation position for comparison. During imaging, the hand was positioned with the palm facing downward alongside the body, and a vacuum-type fixation device was used to secure the hand in both the neutral position and the ulnar deviation position.

For FRACTURE, the inverted black-and-white images were also created to assess whether visibility was affected by color inversion rather than image clarity.

### VR image creation

For FRACTURE, VR images depicting the bones, flexor tendons, and extensor tendons were created using a workstation (Ziostation2 Plus, Ziosoft Inc.) (Fig. 1b).

### Evaluation methods

Tendon visualization was assessed qualitatively and quantitatively.

#### I. Qualitative evaluation

Two orthopedic surgeons (with 21 and 7 years of experience) and two radiologists (with 14 and 6 years of experience) scored the clarity of cross-sectional tendon and VR images.

**Fig. 2:**
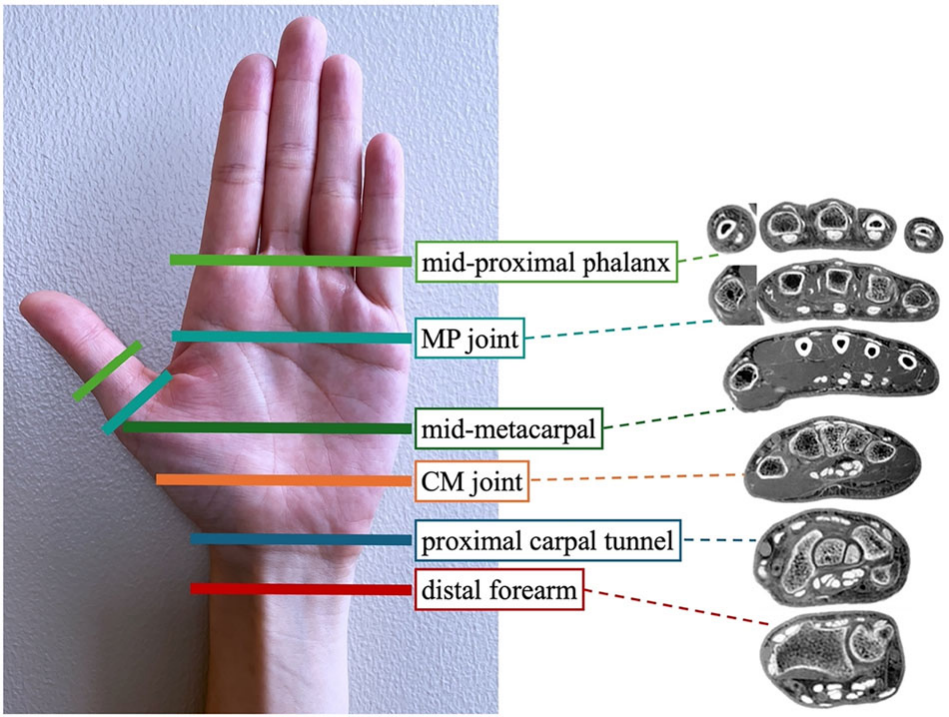
Definition of evaluation levels: evaluation was performed on 24 tendons in total, comprising finger flexor and extensor tendons, using axial images at six locations as illustrated in the figure. An example of a cross-sectional image is a FRACTURE image. Distal forearm, proximal to the distal radioulnar joint where Lister’s tubercle is visible; proximal carpal tunnel, the attachment site for the transverse carpal ligament to the pisiform bone; carpometacarpal (CM) joint, distal carpal tunnel immediately distal to the hook of the hamate; mid-metacarpal, close to the midpoint of the 4th metacarpal bone; metacarpophalangeal (MP) joint, at the thumb MP joint for the thumb and the ring finger MP joint for other digits; and mid-proximal phalanx, at the midpoint of the proximal phalanx in the thumb and the midpoint of the ring finger’s proximal phalanx in the other digits

I-1. Cross-sectional tendon image scoring: the 24 flexor and extensor tendons were evaluated at six levels (Fig. 2). At each level, multiple sequences were evaluated for both neutral and ulnar deviation positions. Scoring was done on a 5-point scale (4: Clearly depicted (sharp edges), 3: Clearly depicted, but a part of the outline is slightly unclear (there are some parts where the boundary line is not clearly visible), 2: Partially unclear outline (unclear parts are less than 50%), 1: Partially unclear outline (unclear parts are more than 50%) (recognizable but not distinct), 0: Not visible (completely unrecognizable)).

Before performing the actual interpretation evaluation, a training session for scoring was conducted with all participants, including two orthopedic surgeons and two radiologists, using one case (204 evaluation points with 4 sequences), separate from the measured cases. Then, for one case, all four participants (two orthopedic surgeons and two radiologists) performed interpretation and scoring. From these results, inter-observer error was calculated, and if the error was determined to be small, a single reader scored the remaining nine cases.

To investigate the magic angle effect, the angle between poorly visualized tendons and the static magnetic field was measured.

I-2. VR image scoring: the clarity of 24 flexor and extensor tendons in FRACTURE VR images was scored on a 3-point scale (2: clearly depicted with tendon continuity observed, 1: some parts of the tendon continuity are interrupted, and 0: not visible (completely indiscernible)). For image interpretation, the following guidelines were established: Overlapping, invisible tendon areas were excluded. For anatomical variations with two tendons, the lower score was assigned. The reading range extended to the joint level proximal to each tendon’s insertion point, excluding the insertions themselves. This is because tendon thinning at the attachment sites causes signal contamination from adjacent tissues, making accurate evaluation difficult and preventing reliable measurements.

Before the interpretation evaluation, all four participants conducted training for scoring. Subsequently, all evaluators scored one case to assess inter-rater reliability before proceeding with single-reader scoring for the remaining cases.

#### II. Quantitative evaluation

II-1. Tendon cross-sectional area difference measurement: for tendon slices that were scored 4 in both FRACTURE and control sequences (T2WI, PD-VISTA), the cross-sectional areas were measured. Since FRACTURE and FRACTURE inversion are identical images with inverted contrast, the comparison targets were limited to T2WI and PD-VISTA. The following comparisons were made: (a) FRACTURE vs PD-VISTA, (b) FRACTURE vs T2WI, and (c) T2WI vs PD-VISTA. The sample size for comparative analysis was calculated using G-power (power = 0.9, effect size = 0.3, *p* = 0.05).

**Fig. 3:**
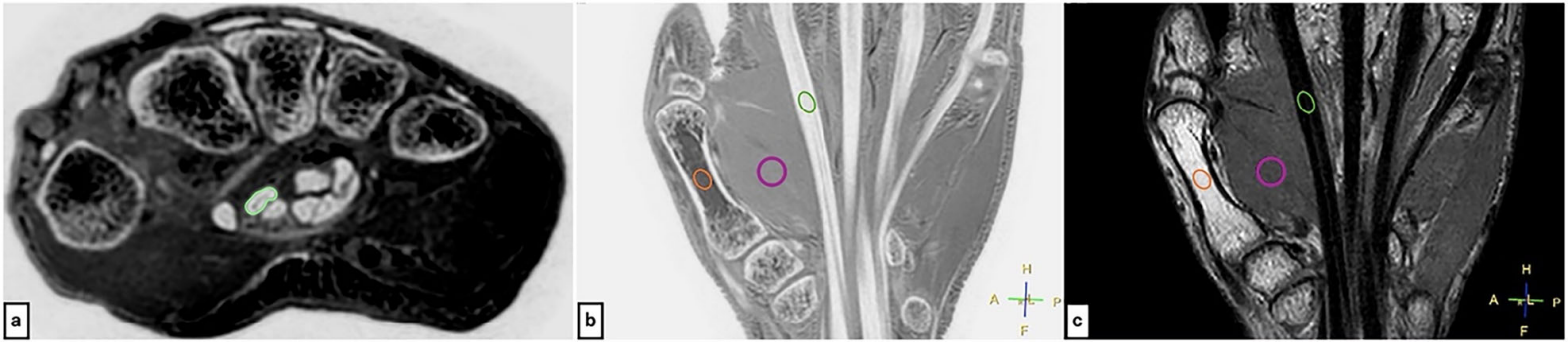
ROI placement diagram: **a** Example of ROI placement (yellow-green circle) for tendon cross-sectional area measurement (image shows FRACTURE sequence). **b** FRACTURE and (**c**) PD-VISTA sequences, where ROIs are set on tendons, muscles, and bone marrow in coronal reformation images (yellow-green circles: tendons, purple circles: muscles, orange circles: bone marrow)

The measurements were performed by an orthopedic surgeon (with 7 years of experience) using the Pencil regions of interest (ROI) tool in DICOM viewer Horos 3.3.6 (Horos Project) to outline the tendon margins as shown in Fig. 3a. The measurement results were reviewed by a senior orthopedic surgeon (with 21 years of experience) who specialized in hand surgery. In cases of disagreement between the two physicians, remeasurement was performed under the guidance of the senior physician.

II-2. Contrast-to-noise ratio (CNR) measurement: the ROIs were set for the tendons, muscles, and bone marrow on coronal FRACTURE and PD-VISTA images acquired in the neutral position (Fig. 3b, c).

The CNR was calculated using the following formula [10]:

$$\root{{2}}\of{\frac{{{\rm{SIa}}}-{{\rm{SIb}}}}{{{{\rm{SDa}}}}^{2}+{{{\rm{SDb}}}}^{2}}\,}$$

Where SI is the mean signal intensity and SD is the standard deviation within the ROI.

CNR measurement was performed on coronal images to match the FRACTURE acquisition plane. T2WI was excluded because it was acquired as a 2D axial sequence, and coronal reconstruction would result in poor image quality due to anisotropic voxels and missing inter-slice data, compromising measurement reliability.

### Statistical analysis

The scoring results for cross-sectional and VR images were expressed as mean scores with standard deviations calculated from 10 subjects and summarized in tabular format by tendon type. Statistical analysis of the qualitative assessments was performed using an ordinal mixed-effects model with the ordinal package in R (version 4.5.1). Pairwise comparisons were adjusted using the Tukey method, and statistical significance was set at *p* < 0.05. Weighted kappa coefficients were calculated to assess inter-rater and intra-rater reliability, as well as the agreement between FRACTURE and FRACTURE inversion scoring.

For tendon cross-sectional area measurements, three pairwise comparisons were conducted: FRACTURE vs PD-VISTA, FRACTURE vs T2WI, and PD-VISTA vs T2WI. Agreement was assessed using Bland–Altman analysis, and relationships were evaluated using correlation analysis.

For CNR analysis, paired *t*-tests were used to compare three tissue pairs (muscle-tendon, tendon-bone marrow, bone marrow-muscle).

For both the Bland–Altman analysis and CNR analysis, Bonferroni correction was applied to control for type I error inflation due to multiple comparisons, with the significance level adjusted to α = 0.017 (0.05/3).

## Results

The present study included 10 healthy volunteers (5 men, 5 women) aged 27–55 years (mean: 37.4 ± 9.1years).

**Fig. 4:**
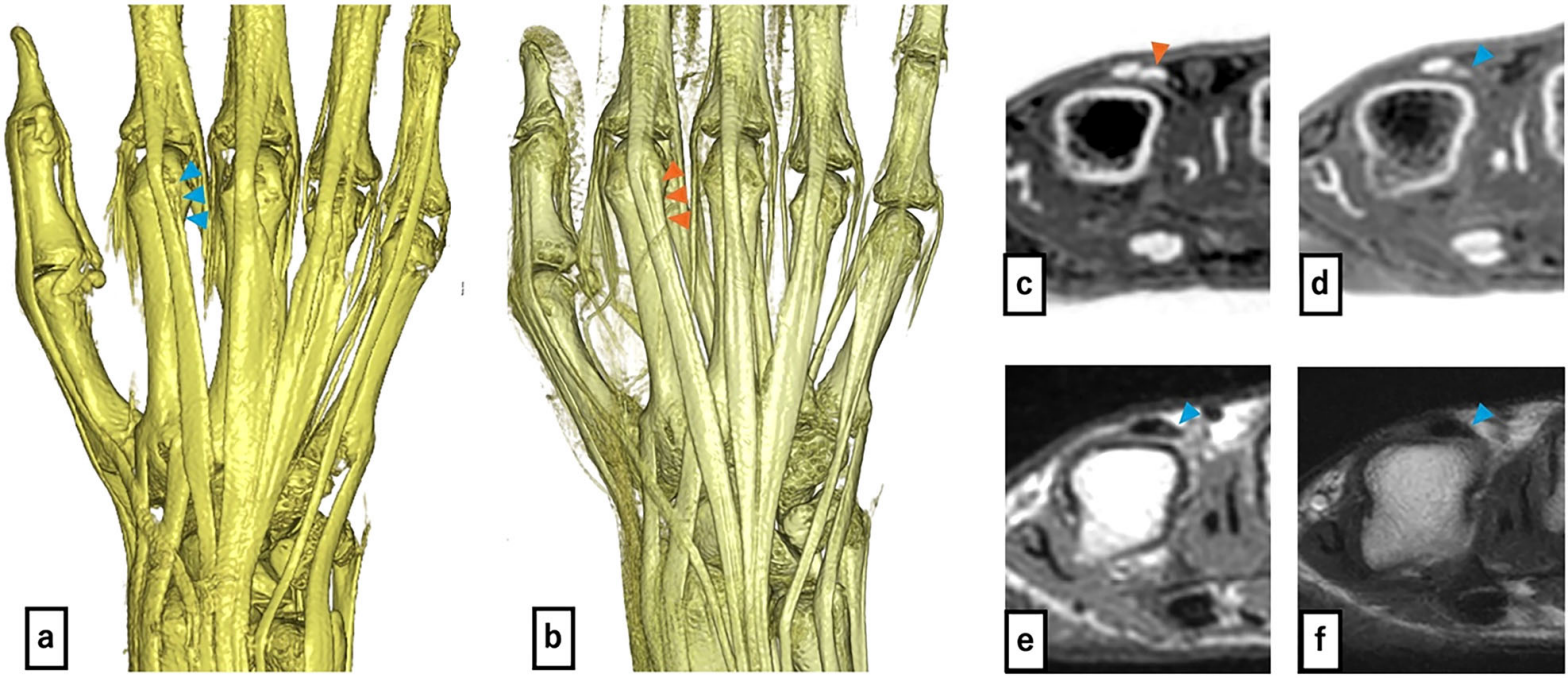
Volume-rendered images from the FRACTURE sequence: (**a**) case with EIP hypoplasia, (**b**) case with normal EIP. Comparison shows the hypoplastic EIP in a (blue and orange triangles: EIP). Axial FRACTURE sequence: (**c**) case with EIP hypoplasia, (**d**) case with normal EIP. **e** Axial PD-VISTA sequence of a case with EIP hypoplasia, (**f**) axial T2-weighted imaging sequence of a case with EIP hypoplasia. The EIP tendon in **c** appears hypoplastic when compared to **d**. The EIP cannot be clearly identified in **e** and **f**, making assessment of hypoplasia not feasible

An important finding was the detection of an anatomical variant (EIP hypoplasia) in one of ten subjects (10%; higher than the reported population prevalence of 0.5–3.5% [2] due to small sample size) (Fig. 4a, d–f).

In the ulnar deviation position, with 0° wrist extension and maximum ulnar deviation, the mean ulnar deviation angle measured from MRI images was 29.69° ± 3.93° (range: 25.9°–37.4°).

### I. Qualitative evaluation

#### I-1. Scoring of cross-sectional tendon images

Initially, all four readers scored one case. Table 2a shows inter-rater and intra-rater reliability results. With inter-rater reliability of κ > 0.4 (moderate agreement or better), the remaining nine cases were scored by a single orthopedic surgeon (intra-rater reliability: κ > 0.7, substantial agreement or better). Table 3 shows the mean and standard deviation of scores for each sequence in 10 cases at the neutral position, and Table 4 shows those at the ulnar deviation position. For detailed score averages, see the Electronic Supplementary Materials. In the neutral position, examining the mean scores for each tendon, most tendons (22 out of 24) showed higher mean scores in the order of FRACTURE and FRACTURE inversion ≧ PD-VISTA > T2WI. However, flexor pollicis longus (FPL) and extensor pollicis longus (EPL) tendons showed lower values for FRACTURE and FRACTURE inversion. Notably, the FPL tendon in FRACTURE and FRACTURE inversion had extremely poor scores from the mid-metacarpal to the metacarpophalangeal joint (MP joint) level. The mean FPL tendon angle relative to the imaging axis in this region was 51.7° (range: 45.5°–53.7°). In ulnar deviation, these tendons showed higher mean scores for FRACTURE and FRACTURE inversion compared to other sequences. Conversely, the extensor digiti minimi (EDM) tendon and flexor digitorum profundus (FDP) tendon of the 5th digit, which were relatively well-visualized in the neutral position, presented with deterioration in scores in the ulnar deviation position. This trend was observed across all sequences. Nevertheless, it was more evident in FRACTURE and FRACTURE inversion.

**Table 2:** Intra-rater and inter-rater reliability (weighted kappa coefficients)

	Surgeon A	Surgeon B	Radiologist C	Surgeon A	Surgeon B	Radiologist C	Surgeon A
	Surgeon B	Radiologist C	Surgeon A	Radiologist D	Radiologist D	Radiologist D	Surgeon A’
(a) Cross-sectional tendon image							
FRACTURE (95% CI)	0.734 (0.595–0.873)	0.515 (0.361–0.669)	0.519 (0.394–0.644)	0.729 (0.607–0.850)	0.629 (0.461–0.769)	0.525 (0.358–0.691)	0.860 (0.753–0.966)
FRACTURE inversion (95% CI)	0.527 (0.376–0.678)	0.488 (0.324–0.653)	0.646 (0.520–0.772)	0.565 (0.424–0.707)	0.479 (0.313–0.645)	0.630 (0.466–0.795)	0.728 (0.620–0.836)
T2WI (95% CI)	0.709 (0.596–0.822)	0.759 (0.662–0.856)	0.601 (0.459–0.743)	0.613 (0.469–0.756)	0.762 (0.663–0.861)	0.659 (0.543–0.775)	0.816 (0.732–0.900)
PD-VISTA (95% CI)	0.663 (0.541–0.784)	0.635 (0.511–0.760)	0.693 (0.575–0.810)	0.577 (0.424–0.730)	0.418 (0.256–0.580)	0.673 (0.542–0.805)	0.711 (0.602–0.821)
(b) VR image							
Neutral (95% CI)	0.846 (0.725–0.967)	0.647 (0.13–0.967)	0.647 (0.13–1.281)	0.647 (0.13–1.281)	0.647 (0.13–1.281)	1.000 (1.000–1.000)	0.778 (0.362–1.193)
Ulnar deviation (95% CI)	0.895 (0.694-–1.096)	1.000 (1.000–1.000)	1.000 (1.000–1.000)	0.895 (0.694–1.096)	0.895 (0.694–1.096)	1.000 (1.000–1.000)	1.000 (1.000–1.000)

A, B: orthopedic surgeons, C, D: radiologists. A, A’: inter-rater reliability

*95% CI* 95% confidence interval

**Table 3:** Mean scores of cross-sectional tendon image (neutral position)

	FRACTURE		FRACTURE inversion		PD-VISTA		T2WI	
	Mean	SD	Mean	SD	Mean	SD	Mean	SD
FCR	4.0	0.00	4.0	0.00	3.7	0.05	3.3	0.72
PL	3.8		3.6		3.0		2.1	
FCU	4.0		4.0		4.0		3.6	
FPL	3.3	0.88	3.3	0.85	3.5	0.50	2.8	1.1
FDS II	3.6	0.36	3.5	0.37	3.3	0.31	3.0	0.42
FDS Ⅲ	3.7	0.38	3.7	0.37	3.4	0.38	3.3	0.27
FDS Ⅳ	3.5	0.35	3.5	0.39	3.3	0.37	3.2	0.39
FDS Ⅴ	2.9	0.40	2.9	0.42	2.8	0.33	2.2	0.57
FDP II	3.7	0.40	3.6	0.40	3.4	0.37	2.9	0.43
FDP Ⅲ	3.6	0.35	3.6	0.33	3.3	0.26	3.1	0.37
FDP Ⅳ	3.2	0.14	3.1	0.17	3.0	0.42	2.7	0.41
FDP Ⅴ	3.0	0.22	2.9	0.23	2.8	0.32	2.2	0.41
APL	2.7	0.75	2.5	0.55	2.2	0.20	1.9	0.78
EPB	3.1	0.77	3.0	0.77	3.0	0.65	1.7	0.52
ECRL	4.0	0.00	4.0	0.00	3.8	0.15	3.4	0.06
ECRB	3.9	0.10	4.0	0.05	3.7	0.10	3.3	0.17
EPL	2.8	0.74	2.6	0.83	2.9	0.44	2.2	0.84
EIP	2.9	0.14	2.9	0.21	2.6	0.08	2.0	0.34
EDC II	3.5	0.36	3.4	0.37	3.1	0.22	2.6	0.44
EDC Ⅲ	3.5	0.29	3.4	0.30	3.1	0.48	2.8	0.59
EDC Ⅳ	3.1	0.28	3.0	0.18	2.8	0.25	2.5	0.33
EDC Ⅴ	2.3	0.30	2.5	0.34	2.2	0.17	1.9	0.37
EDM	3.5	0.41	3.4	0.45	3.2	0.31	2.5	0.23
ECU	4.0	0.00	3.9	0.10	3.2	0.20	2.8	0.56

*FCR* flexor carpi radialis, *PL* palmaris longus, *FCU* flexor carpi ulnaris, *FPL* flexor pollicis longus, *FDS* flexor digitorum superficialis, *FDP* flexor digitorum profundus, *APL* abductor pollicis longus, *EPB* extensor pollicis brevis, *ECRL* extensor carpi radialis longus, *ECRB* extensor carpi radialis brevis, *EPL* extensor pollicis longus, *EIP* extensor indicis proprius, *EDC* extensor digitorum communis, *EDM* extensor digiti minimi, *ECU* extensor carpi ulnaris

**Table 4:** Mean scores of cross-sectional tendon image (ulnar deviation position)

	FRACTURE		FRACTURE inversion		PD-VISTA		T2WI	
	Mean	SD	Mean	SD	Mean	SD	Mean	SD
FCR	4.0	0.00	4.0	0.00	3.9	0.11	3.6	0.44
PL	3.4		3.6		3.1		1.8	
FCU	4.0		4.0		3.8		3.3	
FPL	3.9	0.15	3.9	0.25	3.8	0.15	3.3	0.35
FDS II	3.6	0.34	3.6	0.33	3.4	0.26	2.6	0.22
FDS Ⅲ	3.6	0.46	3.5	0.50	3.4	0.43	2.6	0.30
FDS Ⅳ	2.8	0.53	2.8	0.58	3.0	0.49	2.1	0.51
FDS Ⅴ	1.9	0.86	1.8	0.90	2.2	0.60	1.5	0.50
FDP II	3.7	0.34	3.6	0.33	3.5	0.27	2.7	0.18
FDP Ⅲ	3.6	0.44	3.5	0.38	3.4	0.39	2.3	0.25
FDP Ⅳ	2.6	0.36	2.3	0.34	2.7	0.40	1.8	0.26
FDP Ⅴ	1.8	0.81	1.8	0.90	2.1	0.52	1.6	0.52
APL	3.6	0.10	3.3	0.00	2.6	0.39	2.2	0.00
EPB	3.9	0.11	3.8	0.27	3.1	0.42	2.8	0.43
ECRL	4.0	0.00	4.0	0.00	3.2	0.67	3.4	0.22
ECRB	3.8	0.15	3.7	0.22	3.2	0.11	3.2	0.00
EPL	3.4	0.56	3.2	0.63	3.0	0.31	2.3	0.56
EIP	3.1	0.17	3.3	0.17	3.0	0.25	2.6	0.21
EDC II	3.4	0.41	3.3	0.52	3.1	0.31	2.6	0.57
EDC Ⅲ	3.3	0.42	3.0	0.60	3.1	0.50	2.2	0.61
EDC Ⅳ	2.5	0.27	2.3	0.30	2.5	0.39	1.9	0.40
EDC Ⅴ	2.1	0.57	1.9	0.59	1.7	0.33	1.3	0.52
EDM	2.3	0.82	2.0	0.87	2.5	0.63	1.6	0.51
ECU	3.2	0.60	2.8	0.78	2.8	0.11	2.3	0.78

*FCR* flexor carpi radialis, *PL* palmaris longus, *FCU* flexor carpi ulnaris, *FPL* flexor pollicis longus, *FDS* flexor digitorum superficialis, *FDP* flexor digitorum profundus, *APL* abductor pollicis longus, *EPB* extensor pollicis brevis, *ECRL* extensor carpi radialis longus, *ECRB* extensor carpi radialis brevis, *EPL* extensor pollicis longus, *EIP* extensor indicis proprius, *EDC* extensor digitorum communis, *EDM* extensor digiti minimi, *ECU* extensor carpi ulnaris

The mean scores and standard deviations for each sequence (neutral position) were as follows: FRACTURE, 3.30 ± 0.364; FRACTURE inversion, 3.24 ± 0.609; PD-VISTA, 3.09 ± 0.265; and T2WI, 2.60 ± 0.509.

Comparison of clarity among the four MRI sequences using an ordinal mixed-effects model revealed that FRACTURE demonstrated the highest clarity. When FRACTURE was used as the reference, T2WI exhibited the lowest clarity with a large significant difference (estimate = −1.70, SE = 0.10, *p* < 0.001), followed by PD-VISTA, which also showed significantly lower clarity (estimate = −0.65, SE = 0.09, *p* < 0.001). The difference between FRACTURE and FRACTURE inverse was not statistically significant (estimate = −0.17, SE = 0.09, *p* = 0.076). Furthermore, compared to the proximal region (distal forearm), clarity tended to decrease in more distal regions (one-half of the basal phalanx: estimate = −1.38, *p* < 0.001). Analysis of random effects revealed large variability among tendons (variance = 1.70), whereas variability between subjects was very small (variance = 0.008), indicating that individual tendon characteristics influenced clarity, while systematic differences between subjects were minimal.

The weighted kappa coefficient between FRACTURE and FRACTURE inversion showed high agreement at 0.975 (95% CI: 0.955–0.979).

For the case with EIP hypoplasia, FRACTURE successfully detected the hypoplastic tendon (visibility score = 2), whereas both T2WI and PD-VISTA failed to visualize the structure (visibility score = 0). This finding demonstrates FRACTURE’s enhanced sensitivity for small anatomical structures. The hypoplastic tendon was excluded from area measurement analyses due to non-visualization on conventional sequences.

#### I-2. VR image scoring

**Fig. 5:**
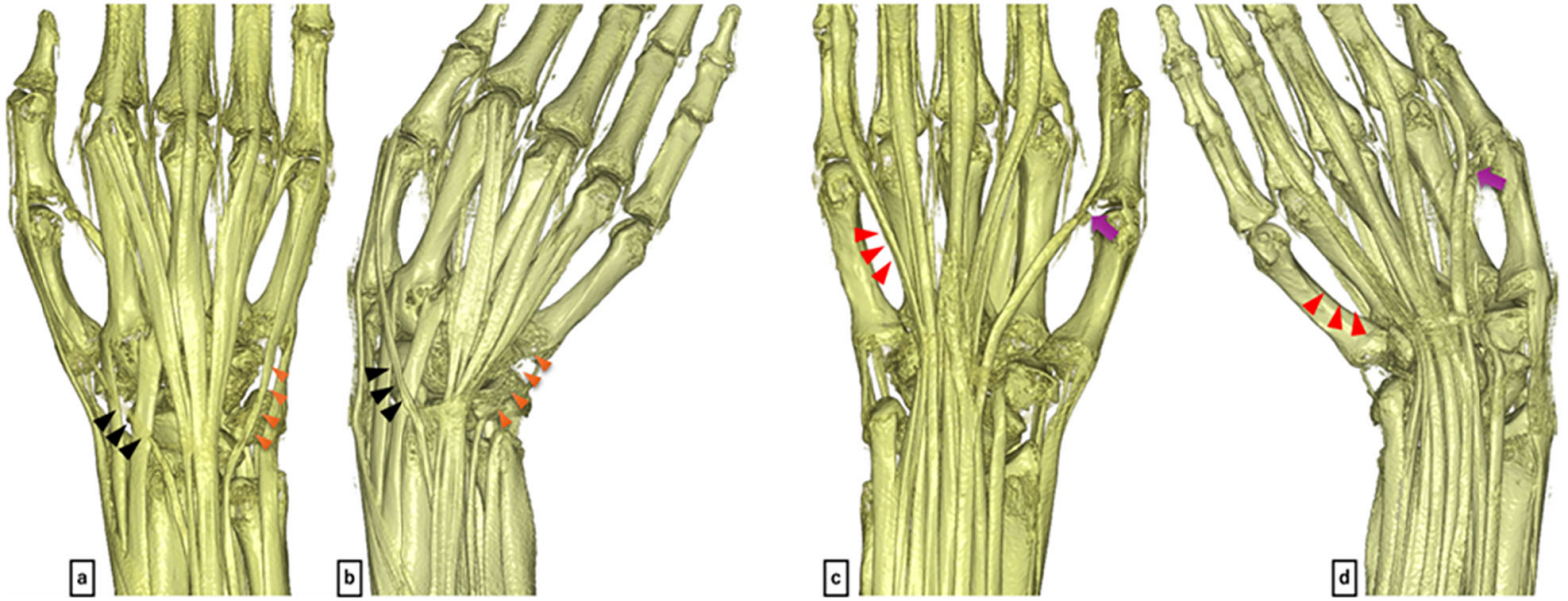
Volume-rendered images from FRACTURE sequence: (**a**) neutral position (dorsal view), (**b**) ulnar deviation (dorsal view), (**c**) neutral position (palmar view), (**d**) ulnar deviation (palmar view). These images demonstrate the impact of the magic angle effect. In the neutral position (**a**, **c**), artifacts are observed in parts of the EPL tendon (black arrowheads) and the FPL tendon (purple arrows), where the tendon course angles relative to the imaging axis were 53.6° for EPL and 53.3° for FPL. In the ulnar deviation position (**b**, **d**), these same areas are clearly visualized. In contrast, the EDM tendon (orange triangles) and FDP tendon of the 5th digit (red triangles), which are clearly visible in the neutral position (**a**, **c**), appear to be absent due to artifacts in the ulnar deviation position (**b**, **d**)

As with the cross-sectional images, all four readers initially scored one case. Table 2b shows the inter-rater and intra-rater reliability results. With inter-rater reliability indicating substantial agreement or better (κ > 0.6), the remaining nine cases were scored by a single orthopedic surgeon (intra-rater reliability: κ > 0.7, substantial agreement or better). Table 5 shows the mean scores for the 10 cases. Additionally, for VR images, the mean score across all evaluated sites was 1.8. The FPL, EPB, and EPL tendons, which had low scores in the neutral position, improved in the ulnar deviation position (with a mean score of 2.0 in the ulnar deviation position). Conversely, the FDP V, EDC IV, and EDM tendons, which had mean scores of 2.0, 1.9, and 1.8, respectively, in the neutral position, decreased to 1.0 in the ulnar deviation position (Fig. 5).

**Table 5:** Mean scores of the VR image

	Neutral	Ulnar deviation
FCR	2.0	1.9
PL	2.0	2.0
FCU	2.0	2.0
FPL	1.4	2.0
FDS II	2.0	2.0
FDS Ⅲ	2.0	2.0
FDS Ⅳ	2.0	1.7
FDS Ⅴ	1.6	1.0
FDP II	2.0	2.0
FDP Ⅲ	2.0	2.0
FDP Ⅳ	2.0	1.8
FDP Ⅴ	2.0	1.0
APL	1.8	2.0
EPB	1.5	2.0
ECRL	2.0	2.0
ECRB	2.0	2.0
EPL	1.4	2.0
EIP	1.9	1.9
EDC II	2.0	2.0
EDC Ⅲ	2.0	1.5
EDC Ⅳ	1.9	1.0
EDC Ⅴ	0.7	0.5
EDM	1.8	1.0
ECU	2.0	1.6

*FCR* flexor carpi radialis, *PL* palmaris longus, *FCU* flexor carpi ulnaris, *FPL* flexor pollicis longus, *FDS* flexor digitorum superficialis, *FDP* flexor digitorum profundus, *APL* abductor pollicis longus, *EPB* extensor pollicis brevis, *ECRL* extensor carpi radialis longus, *ECRB* extensor carpi radialis brevis, *EPL* extensor pollicis longus, *EIP* extensor indicis proprius, *EDC* extensor digitorum communis, *EDM* extensor digiti minimi, *ECU* extensor carpi ulnaris

### II. Quantitative evaluation

#### II-1. Tendon cross-sectional area difference measurement

The sample size for comparative analysis was calculated using G-power (power = 0.9, effect size = 0.3, *p* = 0.05), resulting in 119 slices required for each comparison.

Correlation analysis between tendon cross-sectional area measurements across the three sequences revealed strong positive correlations in all pairs: FRACTURE vs PD-VISTA (*r* = 0.734), FRACTURE vs T2WI (*r* = 0.680), PD-VISTA vs T2WI (*r* = 0.740).

**Fig. 6:**
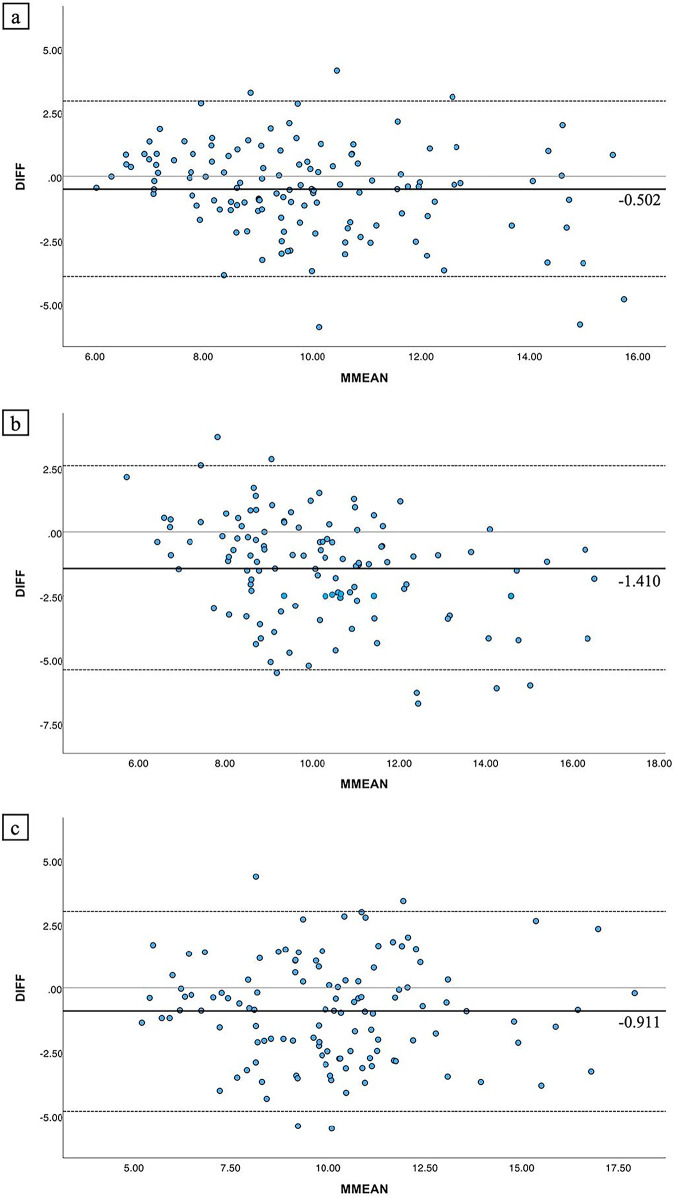
Bland–Altman plots for tendon cross-sectional area measurements. **a** FRACTURE vs PD-VISTA: shows systematic bias with FRACTURE measuring smaller areas (bias = −0.502 mm²; 95% CI: −0.820 to −0.184 mm²). **b** FRACTURE vs T2WI: shows systematic bias with FRACTURE measuring smaller areas (bias = −1.410 mm²; 95% CI: −1.780 to −1.040 mm²). **c** PD-VISTA vs T2WI: shows systematic bias with PD-VISTA measuring smaller areas (bias = −0.911 mm²; 95% CI: −1.273 to −0.548 mm²). Black solid lines represent bias; dashed lines represent 95% limits of agreement

Bland–Altman analysis revealed statistically significant systematic bias between all sequence pairs (all *p* < 0.017, Bonferroni corrected). The bias was −0.502 mm² (95% CI: −0.820 to −0.184 mm²) for FRACTURE vs PD-VISTA (Fig. 6a), −1.410 mm² (95% CI: −1.780 to −1.040 mm²) for FRACTURE vs T2WI (Fig. 6b), and −0.911 mm² (95% CI: −1.273 to −0.548 mm²) for PD-VISTA vs T2WI (Fig. 6c). The 95% limits of agreement ranged from −3.94 to 2.94 mm², −5.40 to 2.58 mm², and −4.82 to 3.00 mm², respectively.

These results indicated that although all sequence pairs showed strong positive correlations, significant systematic bias existed between the sequences.

#### II-2. CNR measurement

The mean CNR values (standard errors) for FRACTURE and PD-VISTA were as follows: For muscle–tendon comparison, FRACTURE: 12.63 ± 1.088 and PD-VISTA: 7.911 ± 1.746 (*p* = 6.29 × 10^−^^5^ < 0.0167). For tendon–bone marrow comparison, FRACTURE: 13.38 ± 4.525 and PD-VISTA: 18.29 ± 5.088 (*p* = 3.44 × 10^−^^2^ > 0.0167). For bone marrow-muscle comparison, FRACTURE: 6.011 ± 3.190 and PD-VISTA: 12.50 ± 2.336 (*p* = 1.81 × 10^-3^ < 0.0167).

FRACTURE showed significantly higher CNR for muscle–tendon comparisons, while PD-VISTA showed significantly higher CNR for bone marrow–muscle comparisons. No significant difference was found for tendon–bone marrow comparisons.

## Discussion

This study demonstrated that the FRACTURE sequence can effectively visualize not only flexor tendons but also extensor tendons and thin finger tendons. In the tendon cross-sectional imaging in neutral position, scoring using a 5-point scale (0–4), flexor tendons showed average scores of generally 3 or higher, and extensor tendons showed average scores of generally 2.5 or higher. Even tendons that are thin and difficult to identify showed average scores above 2. The weighted kappa coefficient between FRACTURE and inverted FRACTURE images was 0.975, indicating excellent agreement. The ordinal mixed-effects model showed no significant difference in visibility scores between FRACTURE and its inverted counterpart. These findings confirm that the superior visualization achieved with the FRACTURE sequence reflects fundamental image quality and tissue contrast rather than subjective reader preference for a particular display polarity or simple contrast differences between black and white presentations.

T2WI is generally considered to have high visualization capability, and previous studies have suggested that PD-VISTA also excels in tendon visualization [13, 14]. FRACTURE provides high-sensitivity imaging of tissues with extremely short T2 relaxation times (short T2 components). Other known techniques for imaging short T2 components include ultrashort echo time (UTE) and zero echo time (ZTE). These techniques directly capture signals from short T2 tissues before signal decay by reducing echo time (TE) to microsecond ranges. The effectiveness of UTE and ZTE has been established for evaluating tendons and ligaments, which are difficult to visualize with conventional methods [15]. Although FRACTURE employs a different approach from UTE/ZTE, it has been reported to demonstrate high concordance with CT in imaging bone, which is also a short T2 tissue [16]. Based on these considerations, the FRACTURE sequence is considered to be effective for tendon evaluation. The ordinal mixed-effects model demonstrated that FRACTURE achieved significantly higher visibility scores compared to PD-VISTA and T2WI. These findings suggest that FRACTURE may provide at least comparable, and potentially superior, visualization of hand tendons compared to conventional sequences. Additionally, the VR images demonstrated favorable visibility, enabling a comprehensive three-dimensional understanding of both flexor and extensor tendons. Based on these findings, FRACTURE can be considered to have potential clinical utility in hand tendon imaging.

The magic angle effect is a phenomenon occurring in short TE sequences, such as FRACTURE, when imaging structures with aligned collagen fibers, such as tendons [17, 18]. This effect causes an increase in the normally low signal of the tendons, thereby making them appear partially absent. The artifacts occur in a cone-shaped area at 54.7° to the static magnetic field direction (B0). This explains the difficulty in distinguishing portions of obliquely oriented tendons, such as the FPL and EPL, in our study. Indeed, referring to the Electronic Supplementary Materials, the FPL tendon had extremely poor scores from the mid-metacarpal to the MP joint level, with a mean angle relative to B0 of 51.7° (range: 45.5°–53.7°). However, not all cases were affected by the magic angle effect. That is, a case with an angle of 45.5° had no effect, and another case with an angle of 53.7° exhibited artifacts. Cautious attention to this phenomenon is important during image interpretation. Both T2WI and FRACTURE had poor scores for the oblique tendons, and comparing the mean scores alone might have indicated similar results. However, in areas where tendons run obliquely, such as the FPL tendon from the mid-metacarpal to the MP joint level or the EPL tendon from the carpometacarpal (CM) joint to the mid-metacarpal level, the FRACTURE scores ranged from 0 to 4. Meanwhile, T2WI consistently scored 1 or 2. This indicated the different underlying causes for poor scores: unclear margins in T2WI vs the magic angle effect artifacts in FRACTURE. To validate tendon presence in affected regions, T2WI (with longer TE) may be necessary, as it is less susceptible to the magic angle effect. Nevertheless, precise quantification of the magic angle effect was technically challenging in our study. Although our FRACTURE and PD-VISTA sequences employ isotropic 3D acquisition, these sequences measure signal intensity based on tissue relaxation properties and do not directly provide collagen fiber orientation information necessary to determine the angular relationship between tendons and B0. The magic angle effect occurs when fibers are oriented at 54.7° relative to B0, but the determination of this angle requires measurement of directional tissue properties. Consequently, quantitative angle-specific CNR analysis was not performed in this study. Future studies employing diffusion tensor imaging, which can map fiber orientation in 3D space, may enable accurate angle-specific CNR analysis and clearer distinction between true pathology and magic angle artifacts.

In our study, the T2WI scores of obliquely running tendons were low, which is thought to be due to the peritendinous tissues, such as tendon sheaths and paratenon, that have similar signal intensity to tendons, making the tendon contours unclear. Furthermore, in cross-sectional images of obliquely running tendons, we believe that the combination of elliptical tendon cross-sections and increased surrounding tissue area may cause the tendon margins to become less distinct on T2WI.

The VR image scoring revealed that tendons like FPL and EPL, which are difficult to visualize in the neutral position, were clearly depicted in ulnar deviation. Conversely, the ring and little finger tendons became less clear in ulnar deviation. This suggests the need to consider imaging position to mitigate the magic angle effect in clinical practice.

The thickness of a tendon is essential information, as hypoplastic tendons may cause dysfunction, and tendons that are too thin cannot be used as donors during functional reconstruction surgery [19]. A key finding of this study was the consistent systematic measurement pattern among sequences: T2WI > PD-VISTA > FRACTURE. FRACTURE consistently measured significantly smaller areas, suggesting that if adequate donor tendon thickness is confirmed with FRACTURE, there is a high likelihood of avoiding the intraoperative discovery of insufficient tendon size. We hypothesize that this systematic difference reflects FRACTURE’s superior ability to distinguish tendons from peritendinous soft tissues compared to T2WI and PD-VISTA, though further research with cadaveric tendons is needed for confirmation.

CNR is an important parameter in MRI that helps evaluate image quality, especially when distinguishing between different tissues, and in previous reports, CNR has been used as a quantitative assessment in depicting wrist structures [20]. In this study, FRACTURE showed significantly higher mean CNR measurements than PD-VISTA in muscle-tendon comparisons. Therefore, FRACTURE demonstrated superior visualization capability for intramuscular tendons.

The EIP tendon, which is an important tendon frequently used in EPL reconstruction, requires caution because there may be anatomical variations that can affect the surgery [21]. There are also reports of cases where, in fact, the absence of EIP was discovered during surgery [19], making accurate preoperative identification highly valuable. In this study, we incidentally detected EIP tendon hypoplasia (Fig. 4a, d, e) compared to a normal EIP tendon image (Fig. 4b, c) using FRACTURE imaging and VR reconstruction. Notably, while FRACTURE successfully visualized the hypoplastic EIP, both T2WI and PD-VISTA failed to detect this structure (visibility score = 0), precluding direct area measurement comparison. This finding demonstrates FRACTURE’s superior detection sensitivity for small or hypoplastic structures rather than systematic measurement underestimation. This capability for detecting anatomical variants that could be clinically important in surgical planning suggests FRACTURE’s clinical utility in hand surgery.

Our study has several limitations. First, the relatively small sample size (*n* = 10) may limit statistical power and generalizability of our results. However, when conducting imaging examinations of healthy volunteers using clinical MRI equipment, ten subjects represented the realistic upper limit due to constraints in equipment availability and volunteer recruitment challenges. Second, while we used T2WI and PD-VISTA as control sequences, future studies should compare FRACTURE with additional imaging sequences to comprehensively assess its clinical utility.

Third, the accuracy of tendon size measurements in FRACTURE images could not be validated due to the absence of a gold standard anatomical reference. Our evaluation was therefore restricted to relative comparisons between imaging sequences. While validation studies using direct cadaveric measurements are currently in progress to compare actual tendon dimensions with both conventional and FRACTURE images, these data were not available for the current study, limiting our ability to assess absolute measurement accuracy. Additionally, our quantitative analysis was limited to completely visualized tendons, potentially introducing selection bias and limiting generalizability to clinical scenarios with suboptimal visualization.

Fourth, our inter-observer assessment was preliminary, being based on a single case. Although this involved the evaluation of 102 tendon cross-sections using four different reading methods across two positions, it may be inadequate for establishing conclusive reliability findings. To address potential expectation bias, we incorporated both standard FRACTURE and FRACTURE inversion sequences into our evaluation protocol. The high weighted kappa coefficient (κ = 0.975) between these methods suggests successful partial mitigation of expectation bias. Nevertheless, limitations remain. The qualitative findings are based on evaluation by a single reader and require independent validation before they can be considered conclusive. Although ordinal mixed-effects modeling demonstrated statistically significant differences, the potential for subjective bias inherent to single-reader assessment cannot be entirely excluded. Additionally, residual expectation bias cannot be completely eliminated due to inherent visual differences between imaging sequences. Future multi-reader studies would strengthen the robustness of these findings.

Finally, this basic investigation in healthy volunteers demonstrates FRACTURE imaging capabilities for normal tendon visualization but does not directly establish clinical diagnostic performance. While we have previously validated the diagnostic utility of FRACTURE imaging and its VR images (including accuracy, reading time, and ease of interpretation) with a small number of readers [22], the current study focused on 3D anatomical visualization quality. Comprehensive validation of VR utility across broader clinical applications, such as preoperative planning, might benefit from larger-scale validation studies (*n* ≥ 20).

In conclusion, FRACTURE and FRACTURE-derived VR imaging were evaluated for tendon visualization in healthy volunteers. FRACTURE is likely to provide clearer visualization of hand tendons compared to T2WI and PD-VISTA. Although FRACTURE is susceptible to magic angle effects, these artifacts can likely be eliminated and a clear visualization achieved by optimizing patient positioning according to the target area. FRACTURE is useful for identifying anatomical variants in normal tendons and may serve as a guide for surgical technique selection, including tendon transfer procedures, potentially making significant contributions to hand surgery practice.

## Supplementary information

ELECTRONIC SUPPLEMENTARY MATERIAL

## Data Availability

The raw data and analyzed datasets obtained from this research will be made available in the Chiba University Repository for Access to Outcomes from Research (CURATOR) (https://opac.ll.chiba-u.jp/da/curator/?lang=0). These datasets contain the minimum data required to verify and reproduce the findings of this study. Due to privacy protection considerations, some data are subject to restricted access.

## Abbreviations

CNR: Contrast-to-noise ratio
EDC: Extensor digitorum communis
EDM: Extensor digiti minimi
EIP: Extensor indicis proprius
EPB: Extensor pollicis brevis
EPL: Extensor pollicis longus
FDP: Flexor digitorum profundus
FPL: Flexor pollicis longus
FRACTURE: Fast field echo resembling a CT using restricted echo-spacing
MP Joint: Metacarpophalangeal joint
PD-VISTA: Three-dimensional proton density-weighted volume isotropic turbo spin-echo acquisition
T2WI: T2-weighted image
UTE: Ultrashort echo time
VR: Volume rendering
ZTE: Zero echo time
