# Supplementary material for: FRACTURE MRI: evaluation of imaging capability in hand tendon visualization using healthy volunteer MRI
Source: Insights Imaging. 2026 Jan 12;17:10. doi: 10.1186/s13244-025-02182-4 (PMC12796028; doi:10.1186/s13244-025-02182-4)
Supplement: Supplementary file 1 — ELECTRONIC SUPPLEMENTARY MATERIAL [file 13244_2025_2182_MOESM1_ESM.pdf]

FRACTURE MRI: Evaluation of Imaging Capability in Hand Tendon Visualization Using Healthy Volunteer MRI

ELECTRONIC SUPPLEMENTARY MATERIAL

a	Neutral position						Ulnar deviation position					
	distal forearm	carpal tunnel	CM joint	one-half metacarpal	MP joint	one-half of the basal phalanx	distal forearm	carpal tunnel	CM joint	one-half metacarpal	MP joint	one-half of the basal phalanx
FCR	4.0	4.0					4.0	4.0				
PL	3.8						3.4					
FCU	4.0						4.0					
FPL	4.0	4.0	3.8	2.0	2.2	4.0	4.0	4.0	4.0	4.0	4.0	3.6
FDS II		3.7	4.0	3.9	3.4	3.0		3.6	3.8	4.0	3.7	3.0
FDS III		3.6	4.0	4.0	3.9	3.0		3.5	3.7	4.0	3.9	2.7
FDS IV		3.8	3.5	4.0	3.3	3.0		3.5	3.2	2.8	2.5	2.0
FDS V		3.6	2.4	2.9	2.7	3.0		3.2	2.2	0.8	1.1	2.2
FDP II		4.0	4.0	3.9	3.4	3.0		3.9	3.9	3.8	3.7	3.0
FDP III		3.6	3.7	4	3.9	3.0		3.9	3.6	4.0	3.9	2.8
FDP IV		3.2	3.1	3.4	3.3	3.0		3.1	2.3	2.8	2.5	2.1
FDP V		3.3	2.7	3.1	2.9	2.8		3.0	1.9	0.7	1.1	2.2
APL	3.4	1.9					3.7	3.5				
EPB	3.7	2.8	3.8	1.9			3.7	3.9	4.0	3.8		
ECRL	4.0	4.0					4.0	4.0				
ECRB	4.0	3.8					3.9	3.6				
EPL	3.8	2.0	1.7	3.2	2.7	3.3	4.0	2.5	3.8	4.0	3.2	3.0
EIP	3.0	3.0	2.9	2.7	2.7		2.9	3.2	3.2	3.0	3.4	
EDC II	4.0	3.7	3.2	2.9	3.7	3.6	3.9	3.7	3.4	3.4	3.6	2.6
EDC III	3.3	3.0	3.9	3.6	3.4	3.7	3.3	2.8	3.9	3.6	3.2	2.7
EDC IV	2.8	2.8	3.2	3.2	3.6	3.0	2.9	2.8	2.6	2.4	2.2	2.2
EDC V	2.7	2.5	2.4	1.8	2.3		2.7	2.6	2.2	1.5	1.3	
EDM	3.9	3.6	3.6	3.8	2.8	3.0	3.8	2.7	1.9	1.5	1.4	2.2
ECU	4.0	4.0					3.8	2.6				

b	Neutral position						Ulnar deviation position					
	distal forearm	carpal tunnel	CM joint	one-half metacarpal	MP joint	one-half of the basal phalanx	distal forearm	carpal tunnel	CM joint	one-half metacarpal	MP joint	one-half of the basal phalanx
FCR	4.0	4.0					4.0	4.0				
PL	3.6						3.6					
FCU	4.0						4.0					
FPL	4.0	4.0	3.8	2.0	2.2	3.8	4.0	4.0	4.0	4.0	4.0	3.3
FDS II		3.5	4.0	3.8	3.2	3.0		3.6	3.7	4.0	3.7	3.0
FDS III		3.6	4.0	4.0	3.7	3.0		3.4	3.6	4.0	3.8	2.6
FDS IV		3.8	3.5	4.0	3.1	3.0		3.6	2.6	2.7	2.3	1.8
FDS V		3.6	2.3	2.8	2.8	2.9		3.2	1.7	0.6	1.2	2.1
FDP II		4.0	3.8	4.0	3.3	3.0		3.9	3.9	3.8	3.7	3.0
FDP III		3.7	3.7	4.0	3.7	3.0		3.6	3.7	3.8	3.8	2.8
FDP IV		3.1	2.9	3.4	3.2	3.0		2.9	2.1	2.4	2.3	1.9
FDP V		3.3	2.7	3.0	2.8	2.7		3.2	1.7	0.6	1.2	2.1
APL	3.0	1.9					3.3	3.3				
EPB	3.2	2.9	3.8	1.7			3.3	4.0	4.0	3.8		
ECRL	4.0	4.0					4.0	4.0				
ECRB	4.0	3.9					3.9	3.4				
EPL	3.4	1.4	1.6	3.1	2.4	3.5	3.8	2.3	3.6	4.0	2.9	2.6
EIP	3.1	3.1	2.8	2.7	2.6		3.0	3.3	3.4	3.2	3.4	
EDC II	4.0	3.7	3.1	2.9	3.3	3.3	3.9	3.6	3.4	3.3	3.4	2.2
EDC III	3.2	3.0	3.9	3.6	3.2	3.5	3.2	2.8	3.8	3.4	3.0	1.9
EDC IV	2.8	2.8	3.1	3.3	2.9	2.9	2.8	2.4	2.4	2.1	2.0	1.9
EDC V	2.7	2.7	2.3	1.9	2.8		2.7	2.2	2.0	1.4	1.0	
EDM	3.8	3.6	3.9	3.6	2.8	2.8	3.8	2.2	1.6	1.3	1.2	1.7
ECU	4.0	3.8					3.6	2.0				

c	Neutral position						Ulnar deviation position					
	distal forearm	carpal tunnel	CM joint	one-half metacarpal	MP joint	one-half of the basal phalanx	distal forearm	carpal tunnel	CM joint	one-half metacarpal	MP joint	one-half of the basal phalanx
FCR	3.7	3.6					4.0	3.8				
PL	3.0						3.1					
FCU	4.0						3.8					
FPL	4.0	4.0	4.0	3.3	2.7	3.2	4.0	3.8	3.8	4.0	3.8	3.6
FDS II		3.3	3.9	3.4	3.1	3.0		3.2	3.7	3.7	3.4	3.0
FDS III		3.2	3.9	3.9	3.2	3.0		3.1	3.7	4.0	3.3	2.8
FDS IV		3.6	3.3	3.9	2.9	3.0		3.4	2.9	3.7	2.3	2.7
FDS V		3.3	2.3	2.6	2.9	2.8		2.9	2.3	1.6	1.4	2.8
FDP II		4.0	3.7	3.4	3.1	3.0		3.6	3.8	3.7	3.4	3.0
FDP III		3.5	3.1	3.7	3.2	3.0		3.4	3.3	4.0	3.3	2.8
FDP IV		3.0	2.5	3.8	2.9	3.0		2.7	2.2	3.4	2.7	2.7
FDP V		3.3	2.3	2.8	2.9	2.8		2.7	2.1	1.6	1.6	2.8
APL	2.4	2.0					2.2	3.0				
EPB	3.3	3.1	3.6	1.9			2.4	3.0	3.3	3.6		
ECRL	3.6	3.9					2.6	3.9				
ECRB	3.6	3.8					3.1	3.3				
EPL	3.4	2.7	2.6	3.4	2.2	3.1	3.3	2.9	3.1	3.4	2.6	2.8
EIP	2.6	2.5	2.7	2.7	2.7		2.6	3.1	3.0	3.0	3.3	
EDC II	3.3	2.8	2.9	3.1	3.3	3.4	3.3	3.2	3.0	3.0	3.3	2.4
EDC III	2.6	2.4	3.8	3.4	3.2	2.9	2.9	2.6	3.3	4.0	3.1	2.6
EDC IV	2.7	2.4	3.0	2.9	3.2	2.7	2.6	2.6	2.8	2.9	1.7	2.4
EDC V	2.3	2.0	2.4	2.0	2.3		2.1	1.7	1.8	1.9	1.1	
EDM	3.0	3.2	2.8	3.8	3.0	3.1	2.9	3.2	2.6	1.9	1.4	3.0
ECU	3.0	3.4					2.7	2.9				

d	Neutral position						Ulnar deviation position					
	distal forearm	carpal tunnel	CM joint	one-half metacarpal	MP joint	one-half of the basal phalanx	distal forearm	carpal tunnel	CM joint	one-half metacarpal	MP joint	one-half of the basal phalanx
FCR	4.0	2.6					4.0	3.1				
PL	2.1						1.8					
FCU	3.6						3.3					
FPL	4.0	3.3	2.6	1.2	1.6	4.0	3.8	3.6	3.3	3.0	3.6	2.8
FDS II		3.7	3.1	2.7	2.4	3.0		2.9	2.3	2.6	2.6	2.9
FDS III		3.3	3.1	3.8	3.3	3.0		2.8	2.6	3.0	2.1	2.4
FDS IV		3.7	2.7	3.7	3.1	3.0		3.0	2.1	1.7	2.2	1.6
FDS V		3.0	2.1	1.2	2.3	2.2		2.0	1.4	0.9	1.0	2.1
FDP II		3.4	3.3	2.4	2.4	3.0		2.9	2.6	2.8	2.4	2.9
FDP III		2.8	2.7	3.7	3.3	3.0		1.9	2.2	2.6	2.4	2.6
FDP IV		2.3	2.1	3.2	2.8	3.0		2.0	1.9	1.6	2.2	1.6
FDP V		2.7	1.8	1.7	2.6	2.0		2.2	1.7	1.0	1.0	2.1
APL	2.7	1.1					2.2	2.2				
EPB	2.4	1.7	1.6	1.0			2.1	3.1	3.2	2.9		
ECRL	3.4	3.3					3.2	3.7				
ECRB	3.4	3.1					3.2	3.2				
EPL	3.1	2.2	1.0	1.6	1.7	3.3	2.4	2.1	2.2	3.3	1.4	2.4
EIP	1.8	1.9	2.6	2.1	1.6		2.4	2.2	2.6	2.8	2.8	
EDC II	3.2	2.8	3.0	2.6	2.0	2.1	3.1	2.8	2.7	2.8	2.7	1.3
EDC III	2.8	2.6	3.9	2.7	2.7	1.9	2.9	2.2	2.6	2.6	2.0	1.0
EDC IV	2.7	2.4	3.1	2.7	2.1	2.2	2.4	2.2	2.1	1.8	1.7	1.2
EDC V	2.4	1.9	2.0	1.7	1.3		2.2	1.6	1.2	0.9	0.8	
EDM	2.8	2.2	2.4	2.9	2.4	2.4	2.7	1.7	1.3	1.3	1.1	1.4
ECU	3.3	2.2					3.1	1.6				

Supplementary Materials 1

Mean scores of cross-sectional tendon image: (a)FRACTURE, (b)FRACTURE inversion, (c)PD-VISTA, (d)T2WI. Scoring is represented by color depth, with darker colors indicating higher scores. These results visually demonstrate that FRACTURE generally achieves superior scores overall.
